# The correlation between attack rates and urban health indicators during the third wave of the COVID-19 outbreak in Turkey

**DOI:** 10.3389/fpubh.2022.986273

**Published:** 2022-11-16

**Authors:** Melike Yavuz, Nilay Etiler

**Affiliations:** ^1^Public Health Department, Bahcesehir University Medical School, Istanbul, Turkey; ^2^Public Health Department, Okan University Medical School, Istanbul, Turkey

**Keywords:** COVID-19, pandemic, attack rates, urban health indicators, spatial analysis, ecologic study

## Abstract

This study aims to analyze the inter-provincial variation in the increase of attack rates in the third wave of the COVID-19 outbreak in Turkey and to determine their relationship with potential urban health indicators. In this ecological study, dependent variables were selected as the COVID-19 attack rates of provinces before the third wave and during the third peak and the attack rate increase ratio. Urban health indicators that can function as determinants of health were calculated for each province under five headings: demographic, health capacity, economic, environmental, and socio-cultural. The epidemiologic maps were produced to show the spatial distribution of COVID-19 attack rates pre- and during the third wave. The associations with urban indicators were conducted using bivariate analysis, including Pearson or Spearman correlation analysis. A multiple linear regression model was run with variables significantly associated with increased attack rates. The results of our study show significant regional variations in COVID-19 attack rates both at the beginning and during the third wave of the COVID-19 pandemic in Turkey. Among the provinces, the attack rate increase ratio has only shown significant correlations to education level and some economic indicators, such as income, employment, industrial activity measured by electric consumption, and economic activity in the manufacturing industry. The multivariate analysis determined that the indicator of economic activity in the manufacturing industry is related to the increase of the attack rate in the third wave. Our results show that the COVID-19 cases are higher in more developed cities with more manufacturing sector activity. It makes us think that it is mainly related to inequalities arising from access to health institutions and testing. It can be determined that the partly lockdown strategy, which excluded the industrial activity in the country, concluded the higher increase in the attack rates in highly industrialized provinces.

## Introduction

The COVID-19 pandemic, starting in December 2019, went down in history as one of the most devastating diseases since the influenza pandemic in 1918. After the COVID-19 epidemic reached the pandemic level, different outbreak control policies were implemented across every country, causing different outcomes in population health (1, 2). The policies of governments to cope with the pandemic emerge from their administrative, economic, and political structures. On the other hand, public adoption of control measures is affected by their demographic, economic, sociological, and cultural characteristics.

**Figure 1:**
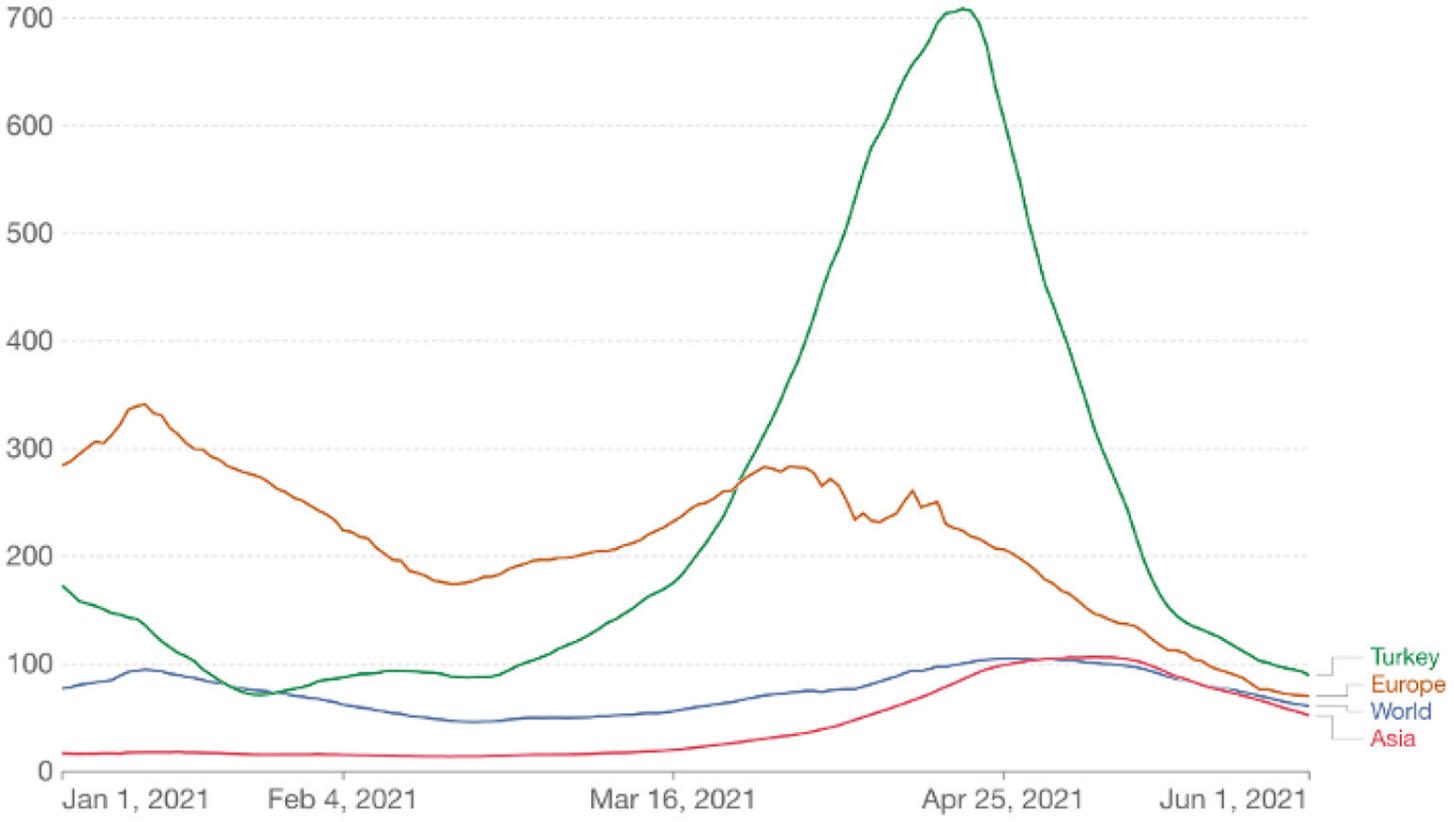
Daily new confirmed COVID-19 cases per million people (7-day rolling average).

Turkey is an extensive and populous country with an area of about 800 km^2^ and a population of more than 85 million. It is subdivided into 81 provinces for administrative purposes. Its economy is classified among the emerging and growth-leading economies, with $815 billion in GDP (3). The COVID-19 outbreak in Turkey started with the first confirmed case on March 11, 2020. Turkey experienced two major epidemic waves in 2020, one peaking in April and the other in December. The third wave, much larger than the previous waves, started in February 2021 and peaked in mid-April (4). Thus, Turkey ranked first in Europe and fourth in the world in the number of cases (Figure 1).

**Figure 2:**
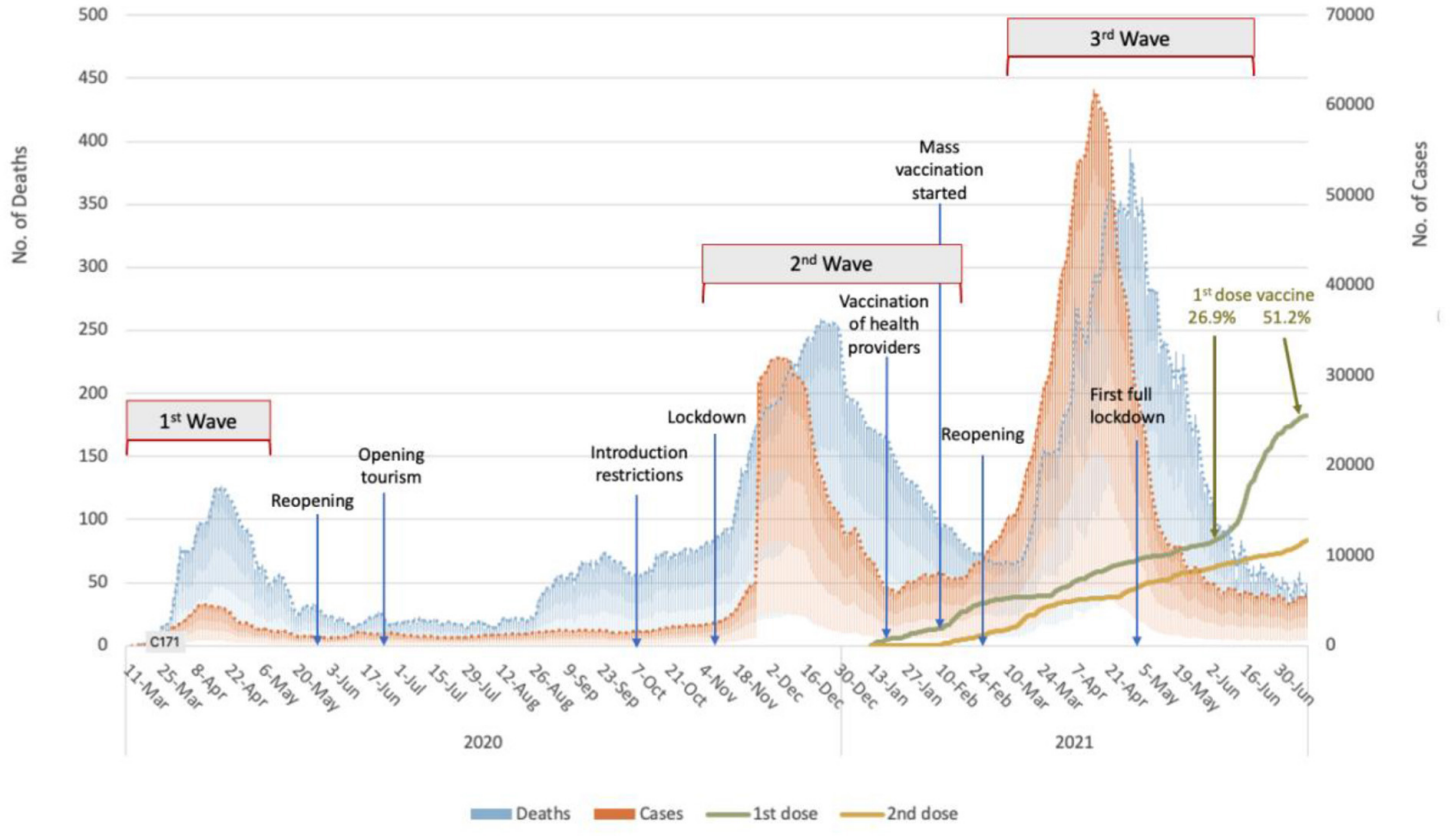
The epidemic curve of the COVID-19 pandemic in Turkey with the lockdowns/openings and vaccination rates between March 2020 to June 2021 (The authors produced the curve to explain the temporal flow).

Figure 2 shows the epidemic curve of COVID-19 in Turkey with the main lockdown and opening times. As understood from the figure, the third wave started just after the second wave's lockdowns reopened. In addition, in this period, only healthcare workers were vaccinated, and widespread vaccination of risk groups had begun but had not yet been completed. Turkey's first and largest lockdown was implemented in the descending phase of the third wave. As shown in Figure 2, although COVID-19 vaccinations began on January 13th, 2021, the vaccination rate was only 26.9% when the third wave ended.

**Figure 3:**
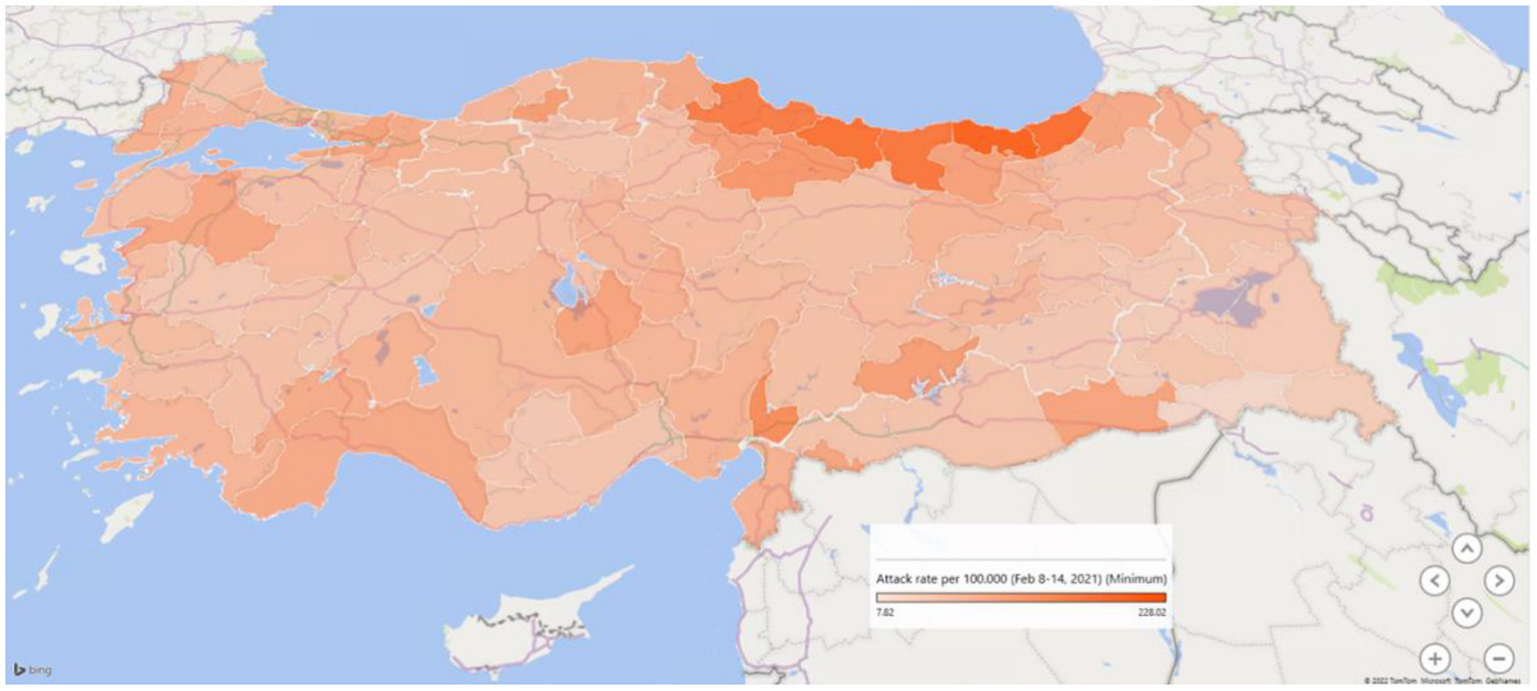
The attack rate of COVID-19 (per 1,00,000 population) on February 8–14, 2021, in the provinces of Turkey.

The third wave affected all provinces in Turkey much more than the first two waves. Although the measures taken to prevent the spread of COVID-19 within the country were generally at the central governmental level, each province was charged with implementation. Thus, there were slight variations in response. During the third wave, incidence and the rate of increase in COVID-19 cases differed remarkably between provinces (Figure 3). The primary motivation of the present study's authors was to investigate why these differences occur. Our study is based on the fact that urban health indicators are crucial in the spread of COVID-19.

The spread of COVID-19 infection shows a spatial distribution that includes the living quarters, micro and macro environment, and social fabric. In the background of this spatial distribution, class characteristics shape the living spaces, environment, and social fabric and even determine the public health problems. It is well known that some people are more at risk of mortality or morbidity from COVID-19 due to where they live and work or their inability to access health services (5). For example, workers in settings such as healthcare facilities, farms, factories, markets, and public transport are naturally at higher risk of exposure to the virus that causes COVID-19 due to the nature of their work. Factors such as discrimination, healthcare access, and use, general health status, education, income, and wealth gaps are associated with more COVID-19 cases, hospitalizations, and deaths (6).

The impact of the pandemic on the populations is also related to the demographic structure, environmental health conditions, capacity of health care, and preventive health services before the COVID-19 pandemic. Population density is considered an essential factor in the spread of COVID-19, as the rate of contact between people is higher in cities with a higher population-weighted density. Several studies have found moderate to strong associations between population density and COVID-19 case and death rates (7–10). It has been suggested that low environmental quality is negatively associated with the human immune system and, as a result, contributes to the COVID-19 pandemic in countries with low environmental quality (11). Previous studies showed a significant negative association between air quality and COVID-19 cases and deaths (12–14). Among air pollutants, PM_2.5_, NO_2_, and O_3_ are reported as the main determinants of COVID-19 (15).

Some researchers have also worked on the socio-economic analysis of the COVID-19 pandemic (16–18). A study conducted in China during the early phase of the pandemic found that cities with more medical resources, as measured by the number of doctors, had lower contagion rates (16). The same study reports that cities with higher GDP per capita have higher transmission rates, which can be attributed to increased social interactions as economic activity increases. Kong et al. (17), in their study examining the relationship between the R0 and socioeconomic parameters, identified that the population between 20 and 34 years old (youth), the people residing in urban agglomerates over 1 million (city), and GINI income inequality have a strong correlation with R0, across countries. An intermediate level of youth, GINI inequality, and a high city population were associated with high R0. A systematic study of the correlations between socioeconomic variables like the GINI index and epidemiological variables like R0 showed a disparity between developed and developing countries and epidemic waves (18).

While some studies regarding the dynamics of COVID-19 in spatial scales, the geographic variation in COVID-19 rates remains to be understood in Turkey, the study by Aral and Bakir (19) on the clustering of outbreak cases on the provincial level in Turkey indicates some environmental, socioeconomic, and healthcare factors. Another ecological study, an analysis of the effects of air pollution on COVID-19 mortality in Istanbul, found that the impact of air quality on COVID-19 emergence interacted with socioeconomic status resulting in a new syndemic (20).

The spatial dynamics of communicable disease outbreaks have been observed to varying degrees, primarily determined by location and influenced by local characteristics. However, this study aims to analyze the inter-provincial variation of the increase of attack rates during the third wave of the COVID-19 outbreak in Turkey and to determine their relationship with potential urban health indicators.

## Materials and methods

This ecologic study analyzes the correlation between the increase in attack rates and some urban characteristics of provinces in Turkey (N: 81). Each province is administered by a governor appointed by the central government and majors whom the residents elect for both metropolitan cities and districts. In each district, there are directories of all ministries of the central government which mirror the national policies on a local scale. However, there are some flexibilities considering the regional dynamic in some cases. The provinces, a fundamental unit of analysis in this study, have their characteristics shaped by their history, economy, demographic, cultural, and social characteristics.

### COVID-19 data

Following the emergence of the first COVID-19 case in Turkey on March 11, 2020, the Ministry of Health officially started to share the data on daily total COVID-19 cases as confirmed by laboratories; thus, the data included only seropositive cases while neither suspicious nor possible cases were reported.

In the current study, the weekly data reported by the Ministry between February 8 and April 30, 2021, were used to calculate the COVID-19 indicators. As shown in Figure 2, the COVID-19 cases began to rise slightly at the end of February 2021, with the number of cases reaching its peak on April 16th at 63,082 new cases. However, the daily case numbers dropped to around 7,000 by May. In the so-called third peak, 2.5 million cases were reported in 3 months, from March 1 to May 31, 2021. However, from the beginning of the outbreak on March 11, 2020, to February 28, nearly 1 year, only 2.7 million cases were reported.

In this study, the COVID-19 data by the province was retrieved from Turcovid19 for analysis (21). The dependent variables of the analysis are:

1. Attack Rate Before the Third Wave (Var1): The number of new cases per 1,00,000 population in the week of February 8th to 14th was accepted as the pre-peak attack rate for each province.
2. Attack Rate During the Third Peak (Var2): Each province's highest attack rate (weekly new cases per 1,00,000 population) was accepted at the peak rate during the third Wave.
3. Attack Rate Increase Ratio (Var 3 = Var2/Var1): It is calculated as a coefficient to estimate the attack rate and how many times increased in the third wave.

### Urban indicators

The indicators which may function as either proximal or distal determinants of health were taken as independent variables in the analysis. These indicators are gathered under five groups: demographic, health care capacity, economic, environmental, and socio-cultural. Table 1 shows the indicators' calculations, data years, and sources.

**Table 1:** The list of urban health indicators.

**Domains/subcategories**	**Indicators of domains**	**Explanations**	**Data source and year**
**Demographic indicators**			
Young people	The proportion of the population younger than 15	[The number of population under 15 years old/total population] × 100	TURKSTAT, Address Based population Registration System, 2019
Elderly people	The proportion of the population 65 aged and older	[The number of the population older than 64 years old/total population] × 100	TURKSTAT, Address Based Population Registration System, 2019
Household size	Average no. of households	It reflects the crowding of houses which is the average number of household members. Crowding is directly associated with infectious diseases and mental health problems.	TURKSTAT, Address Based Population Registration System, 2019
**Health care capacity indicators**			
	Number of medical doctors per 1,000 population	They reflect the pre-pandemic health care system capacity of the province. They can also be used as a measure of the wealth of the region.	TURKSTAT, Health Statistics, 2019
	Number of hospital beds per 1,00,000 population		TURKSTAT, Health Statistics, 2019
**Economic indicators**			
Income	Gross domestic product (GDP) per capita ($)	GDP is the standard measure of the value added created through the production of goods and services in the provinces. The main limitation is being an average for the province but not showing the distribution of it to the people equally.	TURKSTAT, 2019
Deprivation	The proportion of the population under the poverty line according to universal health insurance (%)	It is the rate of poverty according to the Turkish universal health system who has an income of less than one of third of minimal wage besides not having any property. Although it is likely to underestimate all poor people, it is included because of allowing to make an estimation based on province.	Social Security Institution, 2019
Employment	The proportion of insured waged employees with social security (%)	The proportion of the actively employed population under the social insurance system among those older than 15 years old. It reflects employment level.	Social Security Institution, 2019
Industrial activity	Electric consumption (Kwh) of industry per capita	As the industry uses electricity about half of the total amount in Turkey, the indicator is estimated that the consumption of electricity reflects industrialization.	TURKSTAT, Energy Statistics, 2019
Manufacturing production	People employed in the manufacturing industry per 1,00,000 population	[(The no. of workers employed in the manufacturing sector which is from 10 to 32 in NACE codes*)/total Population] × 1,00,000	Social Security Institution, 2019
**Environmental indicators**			
City density	Population density (per km^2^)	Population density per square kilometer	TURKSTAT, Address Based Population Registration System, 2019
Air quality	Concentration of PM10 μg/m^2^	It is included because of being related to emissions of industrial pollution.	Ministry of Environment and Urbanization, 2021
Crowded	The number of students per classroom	It represents public primary schools. The density of classrooms is related to the transmission of communicable diseases among schoolchildren.	TURKSTAT, Child Statistics on Education, 2019
**Socio-cultural indicators**			
Education	Literacy among those older than 15 years old	Literacy is related to development besides is a component of socioeconomic status.	TURKSAT, National Education Statistics, 2020
Gender equality	The proportion of self-employed women (%)	It is calculated using no. of self-employed women by divided to total no. of women (15 yrs+). The rate of self-employed women reflects opportunities for women as an indicator of gender equality.	Social Security Institution, 2019
Social recreation	No. of the saloon for cinema and theater per million population	It is calculated as the total no. of cinemas and theaters by divided t the total population. It is related to social life and social recreation opportunities in the province.	TURKSAT, Cinema and Theater Statistics, 2019
Social networks	No. of society per 1,00,000 population	It is related to networks and civil society capacity.	Ministry of Internal Affairs, 2019

^*^NACE (Nomenclature of Economic Activities) is the European statistical *classification* of economic activities.

The urban health indicators were used after standardization based on ranking the values (22). This method is widely used in urban health studies, especially in building urban health index (23). In the present study, we used each indicator rather than combining them with an index because the direction of some associations with COVID-19 is uncertain. Standardization of each urban indicators (I) was carried out using the formula shown below: I_standardized_ = [I_i_ – min^*^(I)]/[max(I) – min^*^(I)]. In the formula, I_i_ is the mean value of an indicator in the provinces, max(I) is the maximum value and min^*^(I) is the minimum value of that indicator in the same province (23).

### Statistical analysis

Descriptive statistics of the weekly attack rates were calculated as median, mean, standard deviation, minimum, and maximum values for 81 provinces. Kolmogorov-Smirnov test was applied to all variables to test the normality. The attack rate before the third wave was not normally distributed (*p* < 0.05), while the distribution of both peak attack rate and increase ratio were normal (*p* > 0.05). The association of increase ratio with urban indicators was conducted using a bivariate analysis such as Pearson and Spearman correlation. A multiple linear regression model was executed with the significant variables (*p* < 0.10). The analyses were conducted in IBM SPSS 22.0 Program. The epidemiologic maps were produced using Microsoft Excel 2D and 3D maps to show the spatial distribution of both pre-and during the third wave.

## Results

Figure 3 shows a map of the provinces' COVID-19 attack rate (per 1,00,000 population) just before the third wave started in Turkey in February 2021. While the rates were in a wide range between 4.7 and 241.5 per 1,00,000 population, the six provinces in the Region Black Sea had the highest rates ranging between 166.5 to 301.7 per 1,00,000 population.

**Figure 4:**
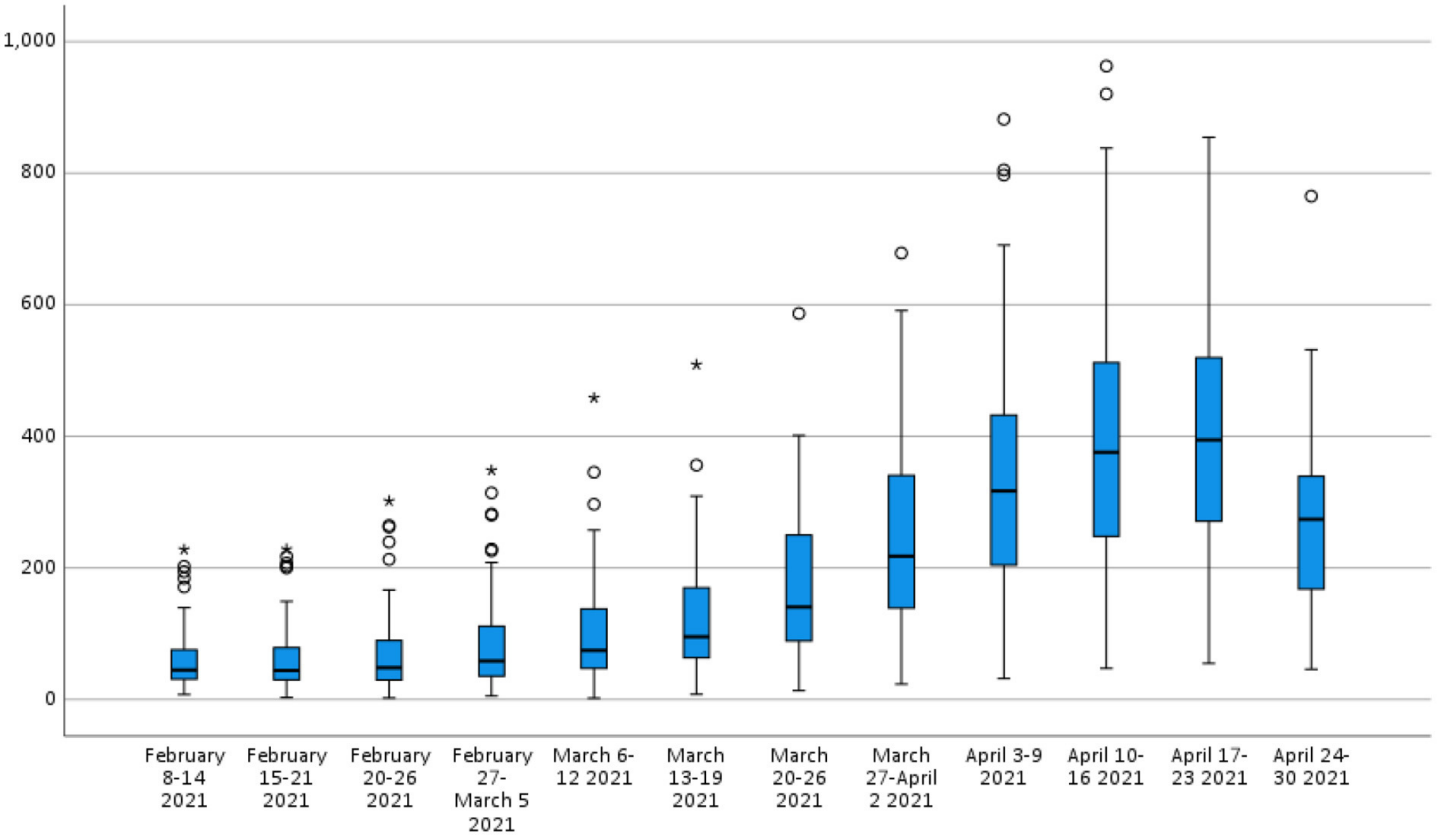
The attack rates of COVID-19 (per 1,00,000 population) by the provinces in Turkey.

Table 2 shows the descriptive statistics of the attack rates of COVID-19 (per 1,00,000 population) in the provinces from February to April 2021 in Turkey. While the attack rates of COVID-19 were statistically similar in the first two weeks (*p* > 0.05), a significant increase started by the third week (February 20–26) (Figure 4, *p* = 0.005). Of the provinces, four (4.9%) reached their peak in the 9th week, 25 (30.9%) in the 10th week, and 52 (64.2%) in the 11th week, April 17-23. Using February as the baseline level, the attack rate increase ratio was between 1.05 and 29.5 times among the provinces.

**Table 2:** Descriptive statistics of the attack rates of the COVID-19 (per 100.000 population) by the provinces from February to April 2021 in Turkey.

**Weeks**	**Date**	**Mean ±SD**	**Median**	**Min. - Max**.
1	February 8–14, 2021	59.0 ± 44.7	44.7	7.8–228.0
2	February 15–21, 2021	60.8 ± 50.1	44.3	3.2–228.4
3	February 20–26, 2021	69.8 ± 61.7	48.5	2.3–301.8
4	February 27–March 5, 2021	85.1 ± 74.4	58.6	5.4–348.4
5	March 6–12, 2021	99.7 ± 84.2	74.6	2.1–458.5
6	March 13–19, 2021	121.4 ± 91.0	95.3	8.2–509.0
7	March 20–26, 2021	172.4 ± 113.1	140.6	13.6–586.8
8	March 27–April 2, 2021	243.2 ± 146.6	217.9	23.4–678.7
9	April 3–9, 2021	335.2 ± 184.5	317.1	32.2–882.1
10	April 10–16, 2021	396.2 ± 196.5	375.8	47.6–963.0
11	April 17–23, 2021	397.4 ± 174.9	394.4	55.0–854.8
12	April 24–30, 2021	266.4 ± 120.3	274.2	46.1–765.2
Differences between 1^st^ week to the peak		360.1 ± 188.5	348.0	4.43–925.4
Attack rate increase ratio		9.6 ± 5.8	8.7	1.1–29.5

Reporting by provinces starting on February 8. While weekly data was Monday to Sunday in the first two weeks, then, it changed to Saturday to Friday.

Table 3 presents the Pearson/ Spearman's correlation analysis between the attack rates and urban indicators in Turkey's third wave of COVID-19 outbreak. The table shows that the attack rate before the wave, during the peak, and increase ratio were analyzed separately as a dependent variable. Although the attack rates before and during the wave are associated with some urban indicators, the attack rate increase ratio is not correlated with most of the indicators. Among the provinces, the increase ratio in the third wave has only shown significant correlations to education level and economic indicators such as income, employment, industrial activity measured by electric consumption, and economic activity of the manufacturing industry.

**Table 3:** The bivariate correlations between the attack rates and urban indicators in the third wave of the COVID-19 outbreak in Turkey.

		**Attack rate before the wave A**	**Attack rate during the peak B**	**Attack rate increase ratio A/B**
**Demographic indicators**				
Young people	The proportion of the population younger than 15	−0.393*	−0.581*	−0.086
Elderly people	The proportion of the population 65 aged and older	0.420*	0.469*	−0.017
Household size	Average no. of households	−0.317*	−0.591*	−0.073
**Health care capacity indicators**				
	No. of MD per 1,000 population	0.239*	0.324*	0.056
	No. of hospital beds per 1,00,000 population	0.087	0.257*	0.050
**Economic indicators**				
Income	Gross domestic product (GDP) per capita ($)	0.136	0.544*	0.320*
Deprivation	% of the population under poverty according to universal health insurance	−0.265*	−0.591*	−0.106
Employment	% of insured waged workers among 15–64 years population	0.145	0.521*	0.259*
Industrial activity	Electric consumption (Kwh) of industry per capita	0.137	0.378*	0.221*
Production of manufacturing industry	People employed in manufacturing industrial activity per 1,00,000 population	−0.009	0.426*	0.379*
**Environmental indicators**				
City density	Population density (per km2)	0.144	0.313	0.182
Air Quality (PM10)	The concentration of PM10 μg/m	−0.131	−0.181	−0.031
Crowded	The number of students per classroom	−0.257*	−0.235*	−0.005
**Socio-cultural indicators**				
Education	Literacy among older than 15 years old	0.163	0.501*	0.193**
Social recreation	No. of the saloon for cinema and theater per million population	0.354*	0.541*	0.103
Social networks	No. of society per 1,00,000 population	0.373*	0.517*	0.053
Gender equality	% of self-employed women	0.327*	0.394*	0.010

*p < 0.05.

**p < 0.10.

**Figure 5:**
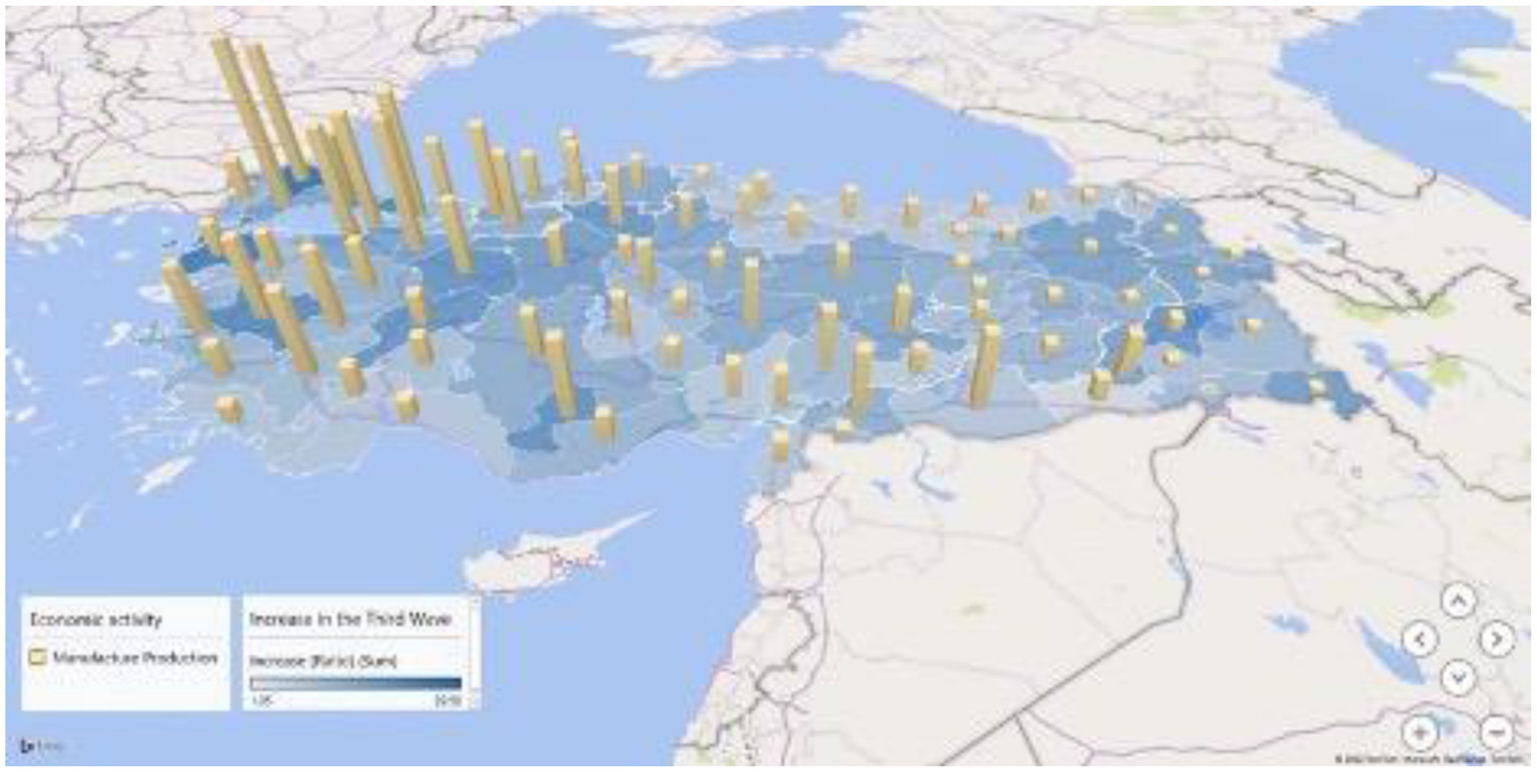
Attack rate increase ratio and manufacturing industrial activity in the third wave of COVID-19 outbreak in Turkey.

We used multiple linear regression analysis to assess the strength of the relationship between the increase ratio of attack rates and several urban indicators, as well as the importance of each of the indicators to the relationship. Since the increase ratio of attack rates from the beginning to the peak of the third wave was normally distributed, we chose the increase ratio as a dependent variable for multiple analyses. In the multiple linear analysis shown in Table 4, the indicator of economic activity in the manufacturing industry showed a correlation to the increase ratio while other variables did not. This association is also presented on the map in Figure 5.

**Table 4:** Multiple linear analysis for the correlates of the attack rate increase ratio in the third wave of the COVID-19 outbreak in Turkey.

**Variables**	**Unstandardized coefficients**		***t***	**Sig**.
	***B***	**Standard error**		
Constant	8.028	1.835	4.376	0.000
Gross domestic product (GDP) per capita ($)	13.084	7.876	1.661	0.101
% of insured waged workers among 15–64 years population	−8.618	7.623	−1.131	0.262
Electric consumption (Kwh) of industry per capita	−3.009	3.800	−0.792	0.431
People employed in manufacturing industrial activity per 1,00,000 population	10.874	4.546	2.392	0.019
Literacy among those older than 15 years old	−0.566	3.771	−0.150	0.881

## Discussion

Our research contributes to the literature in two ways. First, we tested a wide range of urban health indicators at the provincial level during high COVID-19 cases to understand the increases in attack rates in terms of geographic differences. Using urban health indicators enables cross-province comparisons and helps identify associations between health determinants and health impacts. As COVID-19 vaccination started later in Turkey compared to other countries, the third wave in Turkey occurred at a time when vaccination was less common. Thus, the effect of environmental and social factors can be observed as more apparent.

The current study adopts a different approach from existing literature by using provinces as a key unit of analysis. Cities are complex communities of heterogeneous individuals, and multiple factors may be important determinants of health in cities. As Galea and Vlahov (24) indicated, the studies used the city as a critical unit of analysis to reach conclusions about urban characteristics associated with health. This approach also allows us to consider the determinants of health that may be unmeasured since geographical units, such as provinces, have an accumulation of many features in the context of their local dynamics.

Although our study has exciting findings, several limitations exist within our work. First, our study is limited to Turkey. Second, our results are limited to the use of geographically clustered data. Correlations found in ecological studies may not reflect what is observed at the individual level. Furthermore, the shortcomings in data accessibility for more comprehensive analysis should be recognized. Finally, since cases were only laboratory-confirmed, COVID-19 patients that were PCR negative or undiagnosed due to mild illness were not included in the analysis. Caution should be applied when interpreting the results of spatial analytical studies using secondary data sources.

As well-known, individuals older than 65 years are more at risk for both mortality and morbidity of COVID-19 due to higher rates of comorbidity (25). In our analysis, the provinces with a higher proportion of older people were positively correlated with the attack rates. The provinces with a younger population and larger household sizes showed fewer COVID-19 infections. These results were consistent with a recent ecological study examining intraurban variations of COVID-19 incidence in Barcelona. It reported that a high number of neighborhoods with higher numbers of older adults and long-term care facilities were statistically more likely to have a higher number of cases of COVID-19 during the first outbreak of the pandemic (26). An estimated 1% increase in older people or mobility during quarantine would lead to almost 30 extra cases.

The present study found an association between the health care capacity and COVID-19 attack rates. As the number of doctors and hospital beds in the province increased, the attack rates also increased. Expanding access to healthcare facilities can increase the number of cases diagnosed and reduce underreporting, which may contribute to increased coefficients incidence and mortality. A previous study examining the spatial dynamics of the COVID-19 pandemic in Brazil revealed that primary health care coverage is directly related to the incidence of COVID-19 (27). Confirming these findings, a study examining the spatial distribution across African countries found more cases in countries with higher healthcare capacity, as measured by the number of hospital beds and doctors (28). The authors explained this paradox because health care capacity can be used as a measure of a country's wealth. Unlike these results, a recent Malaysian study found no significant relationship between COVID-19 incidence and coverage of primary healthcare services (29). However, the same research explained the low number of cases in regions with low per capita income and a significant GINI coefficient, with insufficient access to health services.

It is noteworthy that all economic indicators under analysis except one showed a correlation with the attack rates in the peak and increase ratios during the third wave. The attack rates during the peak have risen remarkably in provinces with a higher gross domestic product per capita, a higher percentage of insured wage-earners aged 15–64, higher electricity consumption of the industry per capita, a higher number of people per 1,00,000 population in the manufacturing industry and lower population under poverty according to universal health insurance. Our results suggest that the incidence of COVID-19 is higher in more industrialized and economically and financially developed provinces. On the other hand, only manufacturing industrial activity was correlated after applying the multiple regression. A study developed on the estimation and prediction of COVID-19 cases in Brazil confirms the results of our research. It pointed out that the highest number of cases are found in the cities of São Paulo, the country's largest financial and commercial center, and Rio de Janeiro, the most prominent tourist destination (27). Another study from Malaysia observed that the incidence of COVID-19 cases was high in the country's highly industrialized, economic, and financial epicenter (29). These results were consistent with other studies from China (30), India (31), and England (32).

Regarding outbreak control measures in Turkey, industrial activities were excluded from lockdowns, while all other sector activities were highly restricted. The continuation of manufacturing production from the onset of the third wave to the peak may have hindered employees' ability to maintain social distancing as they had to leave home for work, take public transport, and were often exposed to overcrowded conditions. Second, implementing a policy of mass testing by the government in industry and factories, and even in large non-industrial workplaces, may have resulted in significantly higher cases reported. In addition, the low number of cases in provinces with higher poverty populations can be explained by the lack of access to testing opportunities, as argued in previous studies by (28, 29).

Previous epidemiological and spatial analysis studies have revealed that cities with the highest population density also have the highest incidence of COVID-19 (26–29). Contrary, we found no significant correlation between the attack rate of COVID-19 and population density, neither at the beginning of the wave nor at the peak. At first glance, our results seem surprising, given that the virus is spread through human contact and areas with high population density can provide more opportunities for human interactions. It can be explained by several factors that can be confounding. For example, most of the population density may be young people who are less likely to develop symptoms. In addition, both behavioral and policy-induced behavior changes may differ in dense provinces. Studies on the 1918 influenza pandemic have shown that population density may not be correlated with the spread and severity of infectious diseases (33, 34).

Several studies have found an association between air quality and the incidence and severity of COVID-19 (12, 13, 20, 35–37). Atmospheric particulate matter can act as a virus carrier, creating a suitable environment for carrying the virus over longer distances (38). The present study observed no significant relationship between particulate matter pollution and COVID-19 attack rates. With the data obtained from online stations of the Ministry of Environment, Urbanization and Climate Change of Turkey, the annual average PM level of the province was calculated by taking the yearly average of the annual data of all stations in that province. Therefore, the stations may not reflect the air pollution situation of the province throughout the year. Further research needs to be done to establish this relationship by using models that calculate the air pollution of provinces by considering different factors such as prevailing wind, temperature, and humidity.

## Conclusion

The spatial distribution of COVID-19 rates across provinces showed that they did not spread uniformly. The most striking result to emerge from the data is the strong positive correlation between the economic activity of the manufacturing industry and the increase ratio of COVID-19 attack rate in the third wave of Turkey. All our findings support each other, showing that the cases are higher in more developed cities with more manufacturing sector activity. Although greater social mobility can explain this development in cities, it makes us think that it is mainly related to inequalities arising from access to health institutions and testing. According to the results, it can be determined that the partly lockdown strategy, which excluded the industrial activity in the country, concluded the higher increase in the attack rates in highly industrialized provinces.

## Data availability statement

The datasets presented in this study can be found in online repositories. The names of the repository/repositories and accession number(s) can be found in the article/supplementary material.

## Author contributions

All authors listed have made a substantial, direct, and intellectual contribution to the work and approved it for publication.

## Conflict of interest

The authors declare that the research was conducted in the absence of any commercial or financial relationships that could be construed as a potential conflict of interest.

## Publisher's note

All claims expressed in this article are solely those of the authors and do not necessarily represent those of their affiliated organizations, or those of the publisher, the editors and the reviewers. Any product that may be evaluated in this article, or claim that may be made by its manufacturer, is not guaranteed or endorsed by the publisher.
